# An audit of completeness of Road to Health Booklet at a community health centre in South Africa

**DOI:** 10.4102/phcfm.v16i1.4654

**Published:** 2024-12-18

**Authors:** Pfunzo Machimana, Suzan L.N. Nyalunga, Edith N. Madela-Mntla, Doudou K. Nzaumvila

**Affiliations:** 1 Department of Family Medicine and Primary Health Care, Faculty of Health Sciences, Sefako Makgatho Health Sciences University, Pretoria, South Africa; 2 Department of Family Medicine, Faculty of Medicine, University of Pretoria, Pretoria, South Africa

**Keywords:** completion, evaluation, Road to Health Booklet, preschool consultation, Temba

## Abstract

**Background:**

For continuity and quality of care, accurate record-keeping is crucial. Complete care is facilitated by completing a child’s Road to Health Booklet (RTHB) as well as prompt interpretation and appropriate action. This could result in a decrease in child morbidity and mortality.

**Aim:**

The study was aimed at assessing the completeness of the RTHB of children younger than 5 years.

**Setting:**

Temba Community Health Centre (CHC), Tshwane District, South Africa.

**Methods:**

A cross-sectional study was conducted using a data collection sheet adopted from previous studies.

**Results:**

Children less than 1-year-old accounted for 70.2% of the 255 RTHBs. The mean ± s.d. age was 11.5 ±10.76 months. The study finding showed no section was 100% fully completed. Of the 255 records studied, 38 (14.9%) human immunodeficiency virus (HIV)-exposed babies were recorded at birth, 39.5% were negative at 6 weeks and 60.5% were not recorded. Ninety-one (35.7%) children were unexposed. The HIV status of 126 (49.4%) children was not recorded. Sixty-six per cent (66%) of recorded maternal syphilis was negative. Immunisations, weight-for-age, neonatal information, and details of the family and child were fully completed in 80% of the booklets. Developmental screening was 17.2% completed, and oral health was 1.6% partially completed. The overall completeness was 40.3%.

**Conclusion:**

The completeness of RTHBs was found to be suboptimal.

**Contribution:**

The present study’s findings should serve as a reminder that healthcare practitioners must complete RTHBs in their totality in order to improve continuity and care quality, as the results indicated that RTHB completion was below ideal.

## Introduction

Good record-keeping of especially growth data for children is thought to positively impact health outcomes.^1^ Inadequate child development monitoring and the premature mortality of children under 5 years are significant global health concerns.^2^ Childhood is a critical phase, especially in the context of human development, which can have long-term implications on educational and occupation opportunities. Children in low and middle-income nations may be impacted by factors related to socioeconomic status that can hinder access to nutritional food owing to economic constraints.^3,4^ An estimated 5.0 million children under 5 years died globally in 2021 from diarrhoea and malaria, conditions that can be prevented or treated with basic, affordable interventions such as immunisation, proper nutrition, clean water and food, and quality healthcare from trained professionals.^2,5^ Malnutrition inhibits a child’s growth and is one of the major causes of death for children under the age of five. Growth monitoring and promotion (GMP) has been identified as a critical intervention for reducing infant mortality, improving nutritional status and increasing the use of health services worldwide.^6,7^

Government departments and agencies use growth charts to analyse population, formulate policies and execute intervention programmes, as well as to assess a child’s nutritional health, making their completeness and regular audit critical.^8^ This chart mainly served as a record of immunisations and growth monitoring with the goal of enabling earlier and faster diagnosis of abnormal growth curves.^8,9^ This was designed as an easy-to-understand tool that can be used by community health workers as well as to identify children at risk of diseases and to give adherence counselling to the caregivers of those in need of it. The analysis of the collected indirect data suggested a wide range of completeness in Road to Health Card (RTHC) vaccination across Africa.^10,11^ Completion rates vary, posing a challenge for many low-middle-income nations to achieve necessary childhood vaccination coverage.^12^ Road to Health Cards were introduced for use in South Africa in 1973 as part of the strategy to address health promotion in children under the age of 5 years. Addressing recommendations of the charter led to more than 40 different designs being used. In 1987, a new RTHC with a common design was implemented.^11^ Before 2011, the RTHC was an essential monitoring tool for the under-five child health programme. Proper use of the RTHC in primary health care (PHC) was found to improve under-five child health, including growth development.^13^ In 2011, the Department of Health in South Africa replaced the RTHC in maternity facilities and PHC clinics with the Road to Health Booklet (RTHB). Several significant changes have been noted in the new RTHB, which include, among other things, a new format of the three growth charts (Weight for Age, Weight for Height and Height for Age), developmental milestones, oral health, health promotional messages and hospital admission record.^11^ The card mainly served as a record of immunisations and a growth monitoring chart. In 2010, the lack of continuity in human immunodeficiency virus (HIV)-related care prompted the design of a RTHB, which dedicated two pages to HIV-related care.^11,12^ The RTHB also included more detailed growth monitoring, such as head circumference measurements, length-for-age and mid-upper arm circumference (MUAC) and included health promotion messages. Compared with the RTHC, the RTHB serves as a more detailed patient-held child health record and was produced in February 2011.^12,13^

Several challenges have been reported in previous literature. A considerable number of problems relate to poor completion of the length/height × age and weight × length/height growth charts, non-measurement of head circumference, non-completion of the MUAC, developmental screening, deworming and oral health referrals sections, staff shortages and lack of equipment, missed immunisation opportunities, vitamin A non-provision, and no growth monitoring, feeding assessment and provision of nutritional advice.^14,15^

Completing the RTHB is an important aspect that aids in providing essential integrated health services for the growth and well-being of children under 5 years.

The researcher found that healthcare professionals often do not ask for RTHB, interpret it incorrectly, and miss opportunities to identify under-nutrition and immunisation. This study will help plan and implement strategies to promote healthcare services at CHC. The study aimed to assess the current state of RTHB completion for children under-5 years at the Temba Community Healthcare facility.

## Research methods and design

### Study design

This was a cross-sectional study of data extracted from RTHBs of children younger than five attending Temba CHC, from October 2019 to December 2019.

### Setting

This study was conducted at Temba CHC, situated 51 kilometres north of the City of Tshwane located in sub district 2 of the Tshwane Health District in Pretoria, Gauteng province, South Africa.

### Population and sample size

The study population included all RTHBs from children aged younger than five who visit the Temba CHC accompanied by their parents or caregivers. During the period 01 January 2017–31 December 2017, 7656 children visited the facility. An average of 638 children were seen per month. Sample size was based on estimation of the percentage (P) of the RTHBs not well completed.

With a sample size of 246 a two-sided 95% confidence interval for P will be within ±5% of the percentage to be calculated from the sample, if the percentage is of the order of 50%. The sample size calculation was done on nQuery Advance Release 8.0 and was based on the normal approximation of the binomial distribution. The proposed sample size for the study was 241. We oversampled to 255 booklets.

### Data collection

Data were collected by the researcher (P.M.) and a trained research assistant in the field, who trained at Tshwane University of Technology and is a specialist in informatics. Prior to collecting data, the researcher guided the research assistant on the procedures of this study. On the day of data collection, the first parent/caregiver in the queue who consented and provided us with the RTHB, was the starting point of data collection for the day. Thereafter, every third parent/caregiver in the queue was invited to participate in the study when the caregiver/parent was willing. Data were collected three times in a week, namely on a Monday, Wednesday and Thursday. On Tuesdays, there were academic meetings, and not many children presented at the clinic for well-child visits on Fridays. Those who presented did not have the booklets as they came in a hurry because of the child being sick. Data collection was continued until the sample size had been achieved and exceeded by nine more RTHBs. It was assumed that the parent/caregiver presented in a random order at the clinic. The study included all RTHBs of children under 5 years who received child health services at Temba CHC meeting the inclusion criteria. Data were collected from 01 October 2019 until 23 December 2019 for almost a period of 3 months. A modified version of the RTHB was used to collect data adapted from previous studies identified through literature review.^16^

### Data analysis

Data collected were captured on a Microsoft® Excel spreadsheet and exported to SAS, Release 9.4 and then analysed. Data analysis was performed with the assistance of a statistician. The following variables were measured: mother and child’s baseline characteristics; well-child visits; details of the child and family; immunisations; head circumference; neonatal information; mother Prevention of Mother-to-Child Transmission (PMTCT), infant PMTCT information; vitamin A supplementation; deworming; developmental screening; weight-for- age; length/height-for-age; weight-for-height/length and MUAC, clinic visits and oral health examination. Baseline and clinical characteristics of the children were summarised descriptively.

Baseline and clinical characteristics of the children were summarised descriptively. Continuous variables (e.g. age) were summarised by mean, standard deviation, median, interquartile range, minimum and maximum values. Categorical variables (e.g. gender) were summarised by frequency counts and percentage calculations.

The percentage incomplete RTHB was calculated together with a 95% confidence interval. Questions that were found to be usually incompletely filled were identified and reported.

### Ethical considerations

Permission to conduct the study at Temba CHC was obtained from the facility manager. Ethical clearance to conduct this study was obtained from Sefako Makgatho Health Sciences University Research & Innovation Ethics Committee (No. SMUREC/M/66/2019: PG) and the Tshwane Research Committee (TRC) HRD (with reference number GP-201907-03 and project number 46/2019) before conducting the study. Written informed consent was obtained from the parents of the patients in the study. The aim and objective of the study were explained in detail to the parents or caregivers before the information was extracted from the RTHB. Confidentiality and anonymity were maintained throughout the study. No personal identifiers were used.

## Results

Information from 255 RTHBs was recorded. Children below the age of 12 months represented a larger proportion (179; 70.2%) and most of them were female (137; 53.7%). Mean age of the children was 11.5 months with standard deviation of 10.6 month. The youngest child was 1 month with the eldest being 5 years old. The demographic characteristics of the children are presented in Table 1.

**TABLE 1:** Demographic characteristics of children.

Variables	Value					
	*n*	%	Mean	s.d.	Median	IQR
**Age in months**						
≤ 12	179	70.2	-	-	-	-
13–24	50	19.6	-	-	-	-
25–36	16	6.3	-	-	-	-
37–48	7	2.7	-	-	-	-
49–60	3	1.2	-	-	-	-
Total	255	100	-	-	-	-
Mean (± s.d.)	-	-	11.5	± 10.76	-	-
Median (IQR)	-	-	-	-	7	4–18
Minimum/Maximum	1 / 60					
**Gender**						
Female	137	53.7	-	-	-	-
Male	118	46.3	-	-	-	-
Total	255	100	-	-	-	-

s.d., standard deviation; IQR, interquartile range.

Table 2 presents demographic characteristics of the mothers. Most mothers were between the ages of 21 years and 30 years (147; 57.6%). Most children were accompanied by their mothers (211; 82.7%) followed by guardians (39; 15.3%).

**TABLE 2:** Demographic characteristics of the mothers.

Variables	Value					
	*n*	%	Mean	s.d.	Median	IQR
**Age, years**						
14–20	17	6.7	-	-	-	-
21–30	147	57.6	-	-	-	-
31–40	85	33.3	-	-	-	-
> 40	6	2.4	-	-	-	-
Total	255	100	-	-	-	-
Mean (± s.d.)	-	-	28.4	± 5.99	-	-
Median (IQR)	-	-	-	-	28	24–33
Minimum/Maximum	14 / 45					
**Person accompanying**						
Mother	211	82.7	-	-	-	-
Father	5	2.0	-	-	-	-
Guardian	39	15.3	-	-	-	-
Total	255	100	-	-	-	-

s.d., standard deviation; IQR, interquartile range.

Maternal serology completeness is presented in Table 3. Most of the HIV status was not reported (129; 50.6%). It was noted that 169 (66.3%) of mothers had a negative syphilis rapid plasma reagin (RPR) and 86 (33.7%) were not reported. Table 4 presents the completeness of the RTHB at Temba CHC.

**TABLE 3:** Maternal serology completeness.

Variables	*n*	%
**HIV status**		
Positive	34	13.3
Negative	92	36.1
Not completed	129	50.6
Total	255	100
**Syphilis status**		
Positive	-	-
Negative	169	66.3
Not completed	86	33.7
Total	255	100

HIV, human immunodeficiency virus.

**TABLE 4:** Completeness of the Road to Health Booklet at Temba CHC.

Variables		Number (%) of answers							
		Fully completed		Partially completed		Not completed		Not applicable	
		*n*	%	*n*	%	*n*	%	*n*	%
1	Immunisations	243	95.3	12	4.7	-	-	-	-
2	Details of the child and family	205	80.4	47	18.4	3	1.2	-	-
3	Weight-for-age	205	80.4	39	15.3	11	4.3	-	-
4	Neonatal information	204	80.0	48	18.8	3	1.2	-	-
5	Head circumference	165	64.7	16	6.3	32	12.5	42	16.5
6	Vitamin A	138	54.1	9	3.5	17	6.7	91	35.7
7	Well-child visits	123	48.2	130	51.0	2	0.8	-	-
8	Mother PMTCT information	107	42.0	21	8.2	127	49.8	-	-
9	MUAC	87	34.1	56	22.0	67	26.3	45	17.6
10	Deworming	60	23.5	15	5.9	9	3.5	171	67.1
11	Developmental screening	44	17.2	107	42.0	102	40.0	2	0.8
12	Baby PMTCT information	30	11.8	7	2.7	131	51.4	87	34.1
13	Height/length-for-age	27	10.6	97	38.0	131	51.4	-	-
14	Weight-for-height/length	6	2.4	39	15.3	210	82.3	-	-
15	Clinic(s) visited	-	-	4	1.6	251	98.4	-	-
16	Oral health	-	-	4	1.6	162	63.5	89	34.9
	Overall percentage	40.3%		16.0%		30.8%		12.9%	
	95% CI for percentage	38.8–41.8		14.9–17.1		29.4–32.3		11.9–14.0	

PMTCT, Prevention of Mother-to-Child Transmission; MUAC, mid-upper arm circumference.

In 80% of the booklets, the information on immunisations, weight for age, neonatal information and family and child facts were completely filled out. Oral health screening had a 1.6% incomplete rate while developmental screening had a 17.2% incomplete rate. It was 40.3% complete overall.

More than 60% (*n* = 115) of children aged 12 months and younger had 6 scores and less out of 12 for completed items on the RTHB. When compared with children older than 12 months, most children 72% (*n* = 54) in this age group had scores more than 6 (*p* < 0.001). Table 5 represents completions scores of the children. Although there was no association with gender and completion scores, both male and female scores were almost equally proportionate with female having slightly less scores than male.

**TABLE 5:** Road to Health Booklet completion scores.

Characteristic	Completion scores				*P*
	Score less than 6		Score more than 6		
	*n*	%	*n*	%	
**Age group (in months)**					
< 12 months	115	64.3	64	35.7	< 0.001
> 12 months	21	28.0	54	72.0	-
Mean ± s.d. = 6.6 ± 2, Min = 2, Max = 12, Range = 10, Percentile: Lower = 5, Mid = 6, Upper = 8					
**Gender**					
Female	72	52.6	65	47.4	0.44
Male	64	50.0	64	50.0	-

s.d., standard deviation; Min, minimum; Max, maximum; Mid, mid-range.

## Discussion

The majority of the HIV status was not disclosed in almost 50% of the children. Thirty-eight (38) children were exposed (14.9%), while 91 children were not exposed (35.7%). Naidoo discovered that 39.3% of the 56 were HIV-positive (17.6%).^13^

Most mothers in this study (147; 57.6%) were between the ages of 21 and 30. There were only 6 women over 40 (2.4%), while 6.7% of mothers were under 20 (17 mothers). Of the mothers in Ramraj’s study, 70% were women between the ages of 20 and 34. When mothers are there, healthcare professionals have a fantastic opportunity to interact with them about the health and development of their children. Mothers also have a great opportunity to ask the healthcare professional any questions or express any concerns they may have about the child.^17,18,19^

A study conducted at primary, secondary and tertiary levels in Gauteng found a similar result, namely that 85.3% of carers were mothers who brought the child.^20^ Mothers in developing countries are keen to obtain health-related information regarding their child’s health and tend to search frequently for medical information and assistance for their children.^21^ A study conducted in Sri Lanka showed that mothers with a mean age of 28.6 years demonstrated good understanding of their child’s growth pattern and growth charts.

Despite a gradual increase over time, the recording of maternal HIV outcome (67.8%) and maternal syphilis result (69.7%) was very low. Only 50% of the RTHBs had the mother’s HIV outcome reported, and only 6% of the exposed children had their polymerase chain reaction (PCR) findings recorded, which are similar findings of a study from two national facility-based surveys at 6 weeks postpartum.^16^ A significant portion of mothers’ HIV status was unknown (129; 50.6%), 34 were positive and 92 were negative. Ramraj^16^ found that 70% of participants were HIV negative, while 30% tested positive. Poor mother HIV status documentation was shown in Naidoo’s study.^16,11,22^

We noted that 169 women (66.3%) had a negative syphilis RPR, and 86 mothers (33.7%) were not recorded in our study. Despite the fact that it was unclear if Ramraj’s syphilis test results were positive or negative, 67% of them were recorded.^16^

Only 5% of fathers brought their children, while 39% of children were brought by guardians. Most children (211; 82.7%) were brought by their mothers. In a study conducted in Lesotho, the women who comprehend the RTHC used the clinics more for growth monitoring. Children in this trial in Lesotho gained more weight and had a better immunisation record than youngsters who did not utilise the RTHC.^23,24^

According to a Winterveld community survey, mothers brought 76.5% of the children who attended the under-five clinic. According to Tarwa and De Villiers’s study from 2007, women brought the majority of the children. In a similar vein, Ramraj^16^ discovered that 97% of children were brought by their mother.^8,16,17^

The portion on immunisations had the highest percentage of completion (95.3%), which is nearly identical to what other research discovered. The immunisation section, according to Tarwa and De Villiers,^17^ Kitenge and Govender,^25^ Win,^12^ George^7^ and Naidoo,^13^ was thoroughly recorded. Win^12^ discovered that the vaccination section was 100% recorded, which is excellent. As nurses are accustomed to immunising children, it may be why this portion is largely documented.^9,10,11,15,26^ Other research indicates that the incidence of full children vaccines, according to the World Health Organization (WHO) vaccination schedule, is much below the targeted threshold of 90% for reducing childhood illness and mortality, despite the favourable effects on health and the economy. Furthermore, there is significant disparity in immunisation rates among other nations. Vaccination against diverse paediatric diseases is a crucial strategy to attain the Millennium Development Goal of decreasing the mortality rate of children under 5 years old by two-thirds by 2015. Hence, it needs to be regarded as a paramount concern on a worldwide level. This disparity highlights the need for improved and consistent accessibility to healthcare systems in all nations. Only through the analysis of the historical, political and economic factors of each country and the development of measures to overcome specific hurdles to healthcare utilisation can this be achieved. The primary objective is to maximise immunisation rates.^8^

Growth charts are crucial for tracking a child’s development. These (underweight/severely underweight, stunted/severely stunted, wasted/severely wasted, and overweight/obese) aid in determining whether a child is developing normally or not. According to the study, 80.4% of people followed the weight for age growth chart exactly. Similar studies found that more than 80% of the weight for age were fully completed.^7,13,17,22^ The other two growth charts had much lower completion rates than those of the other studies, with 10.6% for height/length for age and 2.4% for weight for height, respectively. The study did not investigate the reasons for not completing this component, which may be because the participants did not understand the significance of the growth charts.

Opportunities were missed to identify the overweight, wasted and stunted. The medical staff had the chance to recognise and refer to severe acute malnutrition while it was still early. According to the RTHB’s instructions, the child’s MUAC is a measurement used to spot malnutrition. From 6 months until the child is 5 years old, MUAC should be assessed at every clinic visit and by community health workers during home visits. Severe acute malnutrition is indicated by MUAC of less than 11.5 cm which requires urgent referral to next level of care. A moderate acute malnutrition index (MUAC) of 11.5 cm – 12.5 cm indicates malnutrition (manage as in Integrated Management of Childhood Illness [IMCI] guidelines) and 12.5 cm or more implies no acute malnutrition (NAM).

The child has severe acute malnutrition if the MUAC is less than 11.5 cm, and moderate acute malnutrition if it is 12.5 cm. The medical practitioner would urgently refer the child to the next level of treatment in such a situation. Our research revealed that 34.1% of MUACs were properly completed, meaning that only 87 of the children may have had malnutrition detected. Even if we combined the fully completed cases with those where age-related MUAC measures were not applicable, the percentage rises to only 51.0%, which is still much less than 80%. This pamphlet was created specifically with that goal in mind to lower morbidity and death.^12,27,28^

In the PHC, malnutrition is frequently discovered after the onset of clinical signs or symptoms, making intervention impossible.^22^ Approximately 57% of the MUAC was completed.^12^

At 14 weeks and 12 months in our study, 64% of RTHBs had fully finished head circumference sections. Our study’s findings outperformed those of other studies which ranged from 3.1% to 58%.^11,12,13,29^

The research showed that 34% of the vitamin A part had been completed. In children aged 12–59 months, this is lower than the Gauteng Province (44.3% in 2013–2014). Moreover, it falls short of the national goal of 60%. Lack of vitamin A can result in both mortality and night blindness.^12,25,26,27,28,29,30^

Deworming treatments should begin at 12 months of age, while vitamin A supplements should begin at 6 months. Afterwards, until 60 months, both should be given every 6 months. This study found that the deworming component was even less completed than the vitamin A section (34%), at only 23.5%. This study’s deworming completion percentage was higher than the University of Pretoria’s (16%), which was only 16%. Nonetheless, it is lower than Win^12^ (52%) and George^7^ (77%). This is even less than the vitamin A completeness.^17,18^

Despite the fact that the frequency of scheduling is roughly the same, deworming is performed far less frequently than vitamin A supplementation, according to the study’s findings. It is most likely a result of a shortage of the deworming drug mebendazole.^12,31^

Early detection of developmental delays can save more issues from occurring and reduce impairment.^14^ Only 17.2% of the section for developmental screening was fully completed, which meant that more than 80% of the children missed out on their development being checked. A higher percentage, 62%, was totally completed in Win’s study.^12^ According to a study conducted in the province of Limpopo, clinic workers failed to complete the developmental screening.^22^ A study in Nepal showed that almost 35% did not meet the Early Childhood Development’s (ECD’s) developmental standards.^2^

The first visit occurs when the first tooth erupts, 6 months after receiving the measles vaccine, and then occurs annually after that. Only four of the booklets were entirely completed, which means that only four children had the chance to participate in an oral assessment. Similar to this, 1% of the booklets in the study by Win^15^ had referrals for oral exams. Cader and Naidoo^32^ indicated a 27% complete rate for the oral health portion, which is still low. Even though the oral health component has been around for almost a decade, this indicates subpar oral healthcare in this PHC. The overall completion mean score for the RTHB was 6.6, which is relatively low. Some of the challenges stated for the lack of completion of the RTHB by healthcare providers were a lack of time, habituation and proper training.^15^ The study’s findings were consistent with earlier studies that observed incomplete data in child monitoring.^6,26,33^

Although the authors’ descriptions of prior studies revealing data omissions involving the RTHB are of scientific interest, it is not possible to compare them directly because the populations and clinics involved in each collection will have been different, and the procedures and circumstances will also have varied. However, this paper adds to prior reports calling for measures to improve completion of RTHB records. Also, the data emphasise the need for greater diligence by clinic staff in this regard to appropriate attention to and use of this standardised health record.

## Conclusion

This study demonstrated that healthcare workers in Temba CHC faced challenges in completing the new RTHB. They managed to complete above 80% of only the following sections: immunisations, weight-for-age, neonatal information and details of the family and child. The completion was so poor that even the socio demographics were not all completed. For example, the booklet would be without the full details of the child, such as the name of the child. It was noted that the healthcare workers had a great challenge in completing the clinic visited and oral health sections. It is necessary to complete the RTHB entirely because it is a crucial book that, when used properly, can lower mortality and morbidity.

### Recommendations

The following recommendations were made based on the findings of the study:

- Healthcare providers need to study guidelines on how to adequately complete the RTHB.
- Structured regular training and retraining of how to complete each section of the booklet.
- Implementation of regular audits of the adequate completion of RTHB.
- Clinic managers should engage with healthcare workers to determine the reasons for poor completion.
- Parents and guardians need to be taught about the importance of each visit and that they should assist in reminding healthcare workers to complete the sections at each visit. This should also include poster at clinics to provide assistance to parents on items that should be completed at each visit.
- A section for parents and caregivers to complete that stipulates that everything that they came for had been completed and, if not, the reason why it has not been completed.
- An app should be developed that will be used by healthcare workers, in which the healthcare workers will have to fill in all the contents needed for that visit – they cannot select what to enter.^18,24^
- To Themba CHC, the management of this clinic must assess its own obstacles and weaknesses in this regard to determine the areas that need to be prioritised in order to improve this part of care.
- To the other clinics, there is a need to review their practices regarding the RTHB. The management must ensure that staff understand the importance of conscientious completion. Also, the management must provide comprehensive training on the use of the RTHB to all staff, and conduct appropriate audits regularly to identify gaps and improve the completeness of data entry.

### Limitations of the study

- The study was conducted only in Temba CHC; therefore, the finding cannot be generalised.
- The study was not conducted every day of the week.
- Certain parents/caregivers refused using the RTHB.
- Some parents/caregivers forgot the RTHB.
- The study is based on a record review of the RTHBs. The inaccuracy and poor completion of the records might affect the quality of the results.
